# Intracranial Complication of Rhinosinusitis from Actinomycosis of the Paranasal Sinuses: A Rare Case of Abducens Nerve Palsy

**DOI:** 10.1155/2014/601671

**Published:** 2014-08-21

**Authors:** G. L. Fadda, M. Gisolo, E. Crosetti, A. Fulcheri, G. Succo

**Affiliations:** ^1^ENT Department, San Luigi Gonzaga University Hospital, Regione Gonzole 10, Orbassano, 10043 Turin, Italy; ^2^ENT Department, Martini Hospital, Via Tofane 1, 10141 Turin, Italy

## Abstract

Sinonasal actinomycosis should be suspected when a patient with chronic sinusitis does not respond to medical therapy or has a history of facial trauma, dental disease, cancer, immunodeficiency, long-term steroid therapy, diabetes, or malnutrition. Radiological evaluation with computed tomography and magnetic resonance imaging are important in differential diagnosis, evaluating the extent of disease, and understanding clinical symptoms. Endoscopic sinus surgery associated with long-term intravenous antibiotic therapy is the gold standard for treatment of sinonasal actinomycosis. We report an unusual case of abducens nerve palsy resulting from invasive sinonasal actinomycosis in a patient with an abnormally enlarged sphenoid sinus. A review of the current literature highlighting clinical presentation, radiological findings, and treatment of this uncommon complication is also presented.

## 1. Introduction

The term actinomycosis derives from the Greek words* aktino*, which refers to the appearance of sulfur granules, and* mikòs*, which defines the condition as a mycotic infection [1, 2]. The annual incidence of actinomycosis is low; in a German study carried out in 2003, its incidence was estimated to be one case per 40,000 inhabitants per year [3].

*Actinomyces israelii* is the primary pathogen responsible bacteria for actinomycosis in humans [4]. It is an anaerobic, slow-growing, and gram-positive organism and is usually a saprophytic and commensal bacterium of the oral cavity [1, 5].

Actinomycosis of the paranasal sinuses (PNS) is very uncommon and has been documented in only a few case reports [1, 5–8]. The most affected region is the maxillary sinus but can involve the ethmoid or sphenoid sinuses [1]. Poor oral hygiene and dental disease have been associated with actinomycosis in the PNS [1, 3, 7, 9]. Actinomycosis in the PNS may complicate with intracranial extension, which clinically presents with headache, visual changes, and cranial nerve palsy [10–12].

Radiological evaluation, as in other sinonasal disorders, is performed with maxillofacial computed tomography (CT) and magnetic resonance imaging (MRI). CT allows the evaluation of the degree of sinus opacification, integrity of bony walls, and presence of calcified lesions or reactive hyperostosis [3]. Moreover, it is useful in the detection of anatomical variations that may predispose to sinusitis [13]. MRI is useful when extension to the orbit, cavernous sinus, optic canal, or other intracranial compartments are suspected, or if malignancy is considered [14].

Long-term antibiotic therapy associated with endoscopic sinus surgery is considered the gold standard for treatment of the disease.

Herein, we report an extremely rare case of invasive sinonasal actinomycosis, involving the maxillary and sphenoid sinuses, that was initially mistaken for a neoplasm. The patient presented with left abducens nerve palsy and diplopia. We also review the clinical and radiological features of sinonasal actinomycosis and suggest appropriate management of this complicated pathology.

## 2. Case Report

A 37-year-old male presented to the emergency department with high fever, severe frontal headache, left retrobulbar and hemifacial pain, and nasal obstruction. The patient had no history of maxillofacial trauma, dental treatment, previous sinus surgery, diabetes mellitus, or other illness.

**Figure 1:**
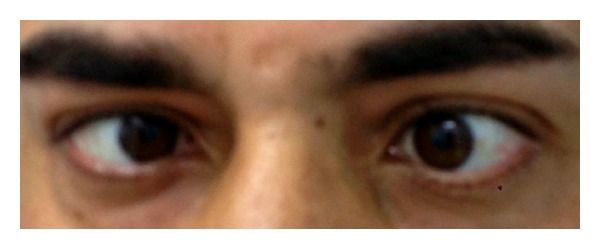
Photo of the patient at admission. Note the absence of motility of the left eye in the lateral gaze related to the deficit of the left lateral rectus muscle for abducens nerve palsy.

Physical examination demonstrated diplopia and absence of motility on left lateral gaze related to deficit of the lateral rectus muscle for abducens nerve palsy (Figure 1). Laboratory tests showed leukocytosis (22.6 × 1000/ul) and an increase in C-reactive protein (14.06 mg/dL). Nasal endoscopy revealed the presence of polypoid neoformations occupying both nasal cavities. Ophthalmological and neurological examinations were negative for other cranial nerve abnormalities, or involving the oculomotor and trigeminal nerves. Visual acuity and pupillary light reflex were also normal.

**Figure 2:**
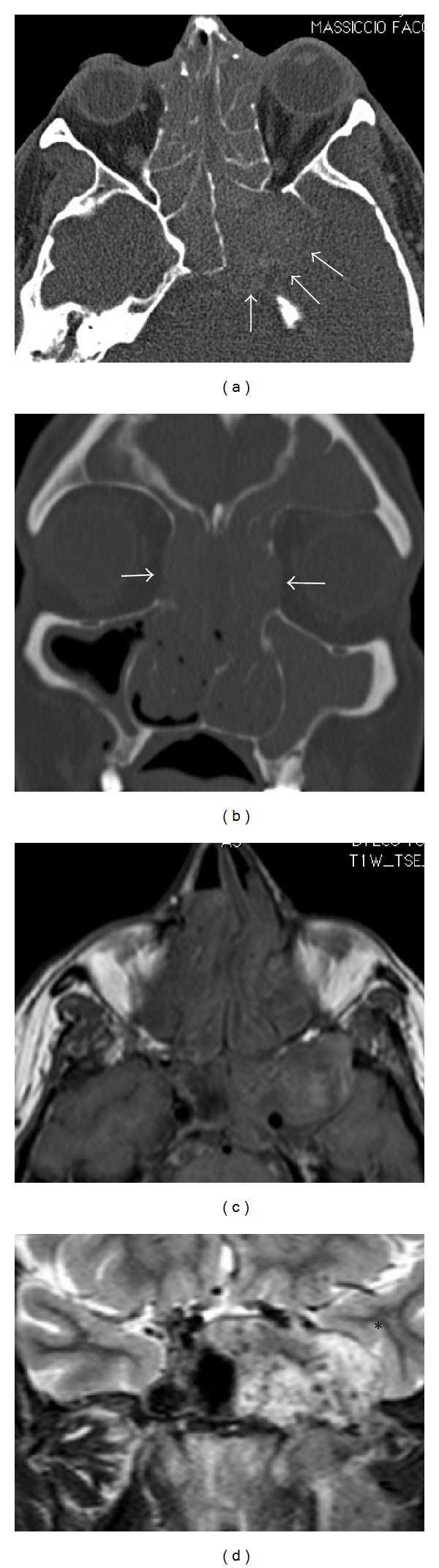
Maxillofacial and brain CT (a, b) and MRI (c, d). (a) Axial CT with contrast medium and (c) MRI-T1 weighted sequence showing abnormal extension of the left sphenoid sinus with thinning of the posterior and lateral bony walls (arrows) and compression of the temporal lobe (asterisk). (b) Coronal CT showing opacification of almost all paranasal sinuses associated with a focal erosion of the papyracea lamina (arrows). (d) Coronal T2-weighted MRI image showing temporal lobe compression (asterisk), while the left abducens nerve and cavernous sinus are not clearly recognizable.

Maxillofacial CT demonstrated a voluminous expansive formation involving the left parasellar region with involvement of the ipsilateral cavernous sinus and all the PNS. Osteostructural rearrangement was present, which was particularly evident in the posterolateral wall of the left sphenoid sinus where it was wider than normal; it was not possible to recognize a well-defined bone structure (Figures 2(a) and 2(b)).

MRI study of the brain, orbits, and paranasal sinuses confirmed the presence of a voluminous left sphenoid sinus; it was occupied by an expansive formation that extended to the left infratemporal fossa, with involvement of the medial and lateral pterygoid muscles and compression of the temporal lobe. It involved the cavernous sinus where the abducens nerve resides, without signs of thrombosis of the internal carotid artery (Figures 2(c) and 2(d)).

Because of these unspecific radiological features, there was no clear preoperative diagnosis, and it was not possible to exclude a neoplasm. The patient was therefore submitted to endoscopic sinus surgery (ESS) with the goal of reducing mechanical compression on the cavernous sinus and inflammation and to obtain a definite histopathological diagnosis. Bilateral transethmoidal sphenoidectomy was performed with extensive removal of the anterior left sphenoid wall. The wide sphenoidectomy allowed visualization of the abnormal extension of the left sphenoid sinus and its lateral recess. There was no bone destruction.

**Figure 3:**
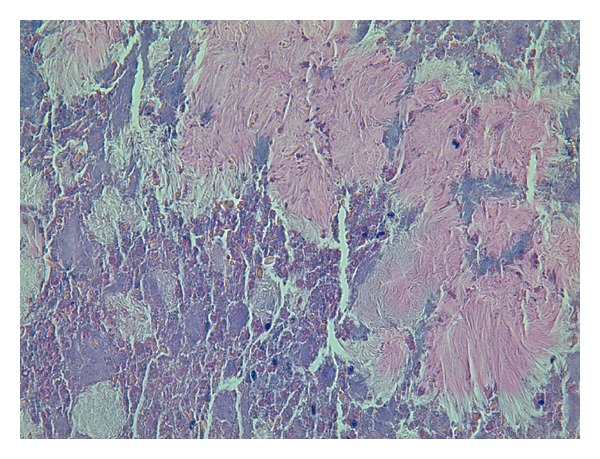
Actinomycetes can be seen forming colonies of filamentous sulfur granules surrounded by numerous polymorphonuclear cells (hematoxylin and eosin staining, 40x).

Histological examination revealed the presence of chronic rhinosinusitis associated with invasive actinomycosis of the left maxillary and sphenoid sinuses (Figure 3). In addition, bacteriological culture and antibiograms from the purulent material collected during the procedure revealed the presence of polymicrobial infection (*Actinomyces israelii*,* Staphylococcus haemolyticus*, and* Citrobacter braakii*).

On this basis, the infectologist recommended two weeks of therapy with intravenous vancomycin (1 g BID), ceftriaxone (2 g BID), levofloxacin (500 mg daily), pantoprazole (40 mg daily), and methylprednisolone (40 mg BID). The patient was discharged and prescribed 8 weeks of therapy with oral amoxicillin-clavulanate (1 g BID) and saline nasal irrigations.

**Figure 4:**
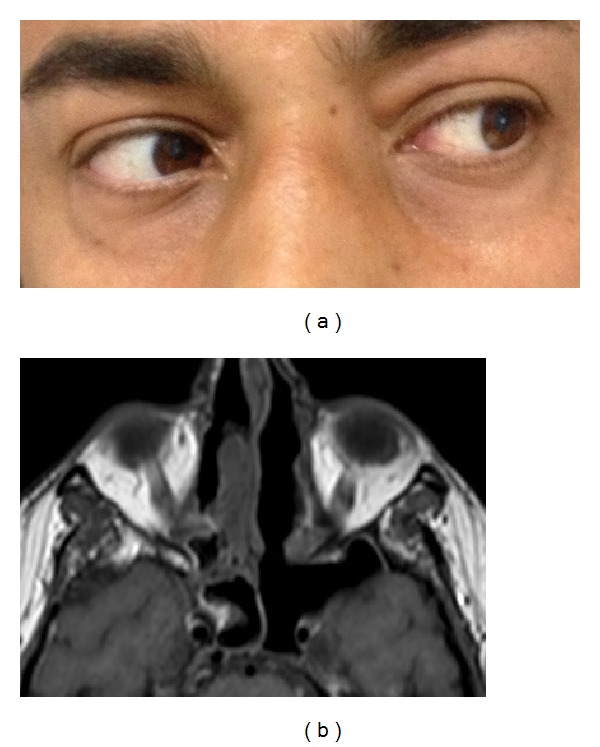
(a) Photo of the patient at three months after ESS showing complete recovery from left abducens nerve palsy. (b) Axial MRI performed one month after surgery. Enlargement of the left sphenoid sinus and resolution of the disease can be seen.

The patient had gradual resolution of all symptoms, and complete recovery from abducens nerve palsy and diplopia occurred at three months after surgery (Figure 4(a)). Brain and maxillofacial MRI was performed one month after surgery and revealed resolution of inflammation and highlighted the abnormally enlarged sphenoid sinus (Figure 4(b)).

Regular endoscopic follow-up has been performed. At one year after ESS, there was no evidence of recurrence of disease with complete resolution of symptoms.

## 3. Discussion

Actinomycosis is a chronic infectious disease with granulomatous and suppurative features. It has three clinical forms: cervicofacial, pulmonothoracic, and abdominopelvic with frequencies ranging 41–55%, 15–34%, and 13–20%, respectively [4, 7, 9, 14]. It is a rare disease, which may explain why there is often little clinical suspicion leading to frequent delays in diagnosis and appropriate treatment [6]. There is a male predominance (1.5–3 : 1) and it generally affects patients from 40–70 years, without racial predilection [7, 8].

Few cases of paranasal sinus actinomycosis have been reported [1, 5–8]. It should, however, be suspected when a patient with chronic sinusitis does not respond to medical therapy or has a history of facial trauma, dental disease, or dental treatment [1, 4]. Other pathogenetic factors include cancer, immunodeficiencies such as HIV, long-term steroid therapy, diabetes, and malnutrition [15].

In uncomplicated actinomycosis involving the PNS, clinical symptoms and signs are not different from those of any other infective sinusitis.

In invasive actinomycosis of the PNS, as in our patient, headache is the most frequent presenting symptom (64−100%) [10–12, 16]. It usually presents with retroorbital and/or vertex localization, hyperalgesia and can be associated with fever. Visual deficit is the second most common presenting symptom [10–12]. In the literature, changes in visual acuity can be found in up to 42% of patients with sphenoiditis [10], while diplopia has been reported in only 15% [12]. The etiology of diplopia is most commonly related to cranial nerve VI palsy, which suggests clivus and/or cavernous sinus involvement [16]. In fact, the abducens nerve (cranial nerve VI) is closest to the sphenoid sinus, and its paralysis is the most common complication in sphenoid sinus disease [11, 17] complicated with intracranial extension, as in this case. Although thrombophlebitis of the cavernous sinus was not present in our patient, it is mandatory to exclude this rare complication by brain MRI.

Radiologic (CT and MRI) findings are not specific in PNS actinomycosis [1, 7]. In the present case, the extreme thinning of the posterior and lateral walls of the left sphenoid sinus demonstrates the tendency of actinomycotic infections to spread without regard for anatomical barriers. Diagnosis of actinomycosis is confirmed by the isolation of* Actinomyces* in culture, histopathological identification of the bacteria on biopsy specimens, or visualization of typical sulfur granules [1, 4, 9, 14]. Nevertheless, it can be difficult to isolate species that are in synergy with other aerobic and anaerobic bacteria [1, 4, 9]. Differential diagnosis includes benign lesions, primary or metastatic tumors, and Wegener's granulomatosis [2].

It remains unclear whether the presence of a large left sphenoid sinus as an anatomical variation may have been associated with predisposition to this complication. While there is very limited evidence in the literature to suggest such an association, this needs to be addressed by further studies. In our opinion, this association is likely.

Treatment of invasive PNS actinomycosis requires a combination of ESS and long-term antibiotic therapy, as reported by many authors [1, 4, 8]. The antibiotic of choice is penicillin, while other studies have shown that tetracycline and clindamycin are acceptable alternatives, especially in the setting of penicillin allergy [1, 5, 18, 19].

In our patient, medical therapy was different, since the presence of polymicrobial infection (*Act. israelii*,* Staph. haemolyticus*, and* Citr. braakii*) required antibiogram-based multidrug therapy.

Because of the reduced vascular supply in the inflamed sinuses, the tissue distribution of antibiotics is limited and antibiotic therapy alone is not effective [4, 15]. Therefore, endoscopic surgical removal of the involved tissues and restoration of sinus ventilation is mandatory for treatment of actinomycosis involving the PNS. After ESS, the duration of intravenous antibiotic therapy varies among reports, but should generally be continued for 2–6 weeks. Oral penicillin is then required for an additional 2–12 months, depending on the severity of disease and response to treatment [18, 19].

Finally, postoperative management is important because recovery from visual disturbances is frequently slow, and long-term follow-up is needed. Improvement in extraocular movements during the first three months following surgery is a good prognostic sign, as observed in our patient; however, the average recovery time from diplopia reported in the literature is 5.1 months [16].

## 4. Conclusion

Actinomycosis of the PNS typically presents with vague symptoms that initially mimic chronic rhinosinusitis, but with more aggressive behavior and bony erosion as long as the infection is present. In the presence of signs and symptoms such as severe headache, visual disorders, and cranial nerve paralysis, both CT and MRI must be performed for correct evaluation of bone integrity and extension of sinonasal inflammatory disease into the orbit, cavernous sinus, or intracranial compartments. However, specific diagnosis of actinomycosis requires microscopic confirmation based on the identification of sulfur granules in culture or histopathology. We emphasize surgical endoscopic removal of the involved tissues to restore good sinus ventilation and long-term intravenous antibiotic therapy as treatment for invasive actinomycosis of the PNS. At last, long-term follow-up is mandatory since recovery from oculomotor palsy progresses slowly after surgery.
